# Antioxidant Enzymes Haplotypes and Polymorphisms Associated with Obesity in Mexican Children

**DOI:** 10.3390/antiox9080684

**Published:** 2020-08-01

**Authors:** Paula Costa-Urrutia, Aline Mariana Flores-Buendía, Iván Ascencio-Montiel, Jacqueline Solares-Tlapechco, Omar Noel Medina-Campos, José Pedraza-Chaverri, Julio Granados, Angélica Saraí Jiménez-Osorio, Martha Eunice Rodríguez-Arellano

**Affiliations:** 1 Laboratorio de Medicina Genómica, Hospital Regional Lic. Adolfo López Mateos, ISSSTE, Ciudad de Mexico 01030, Mexico; paula.costa.urrutia@gmail.com (P.C.-U.); alinefloresbuendia@gmail.com (A.M.F.-B.); jsoltlapechco@gmail.com (J.S.-T.); 2 Departamento de Biología, Facultad de Química, Universidad Nacional Autónoma de Mexico, Ciudad de Mexico 04510, Mexico; omarnoelmedina@gmail.com (O.N.M.-C.); pedraza@unam.mx (J.P.-C.); 3 Coordinación de Vigilancia de Epidemiología, Instituto Mexicano de Seguro Social, 120 Mier y Pesado Street, del Valle Benito Juárez, Ciudad de Mexico 03100, Mexico; ivan-ascencio@hotmail.com; 4 División de Inmunogenética, Departamento de Trasplantes, Instituto Nacional de Ciencias Médicas y Nutrición Salvador Zubirán, Ciudad de Mexico 14080, Mexico; julgrate@yahoo.com

**Keywords:** antioxidant enzymes, polymorphisms, children obesity

## Abstract

Obesity is a major health problem worldwide and constitutes a sanitary emergency in Mexico, especially childhood obesity. Several studies have proved the relationship between obesity and oxidative stress and the influence of genetic predisposition. This work was aimed to analyze the association of antioxidant enzyme polymorphisms with overweight and obesity in Mexican children and adolescents. A case-control study was performed in 585 children and adolescents aged 3 to 17 years, using two criteria to classify obesity: body mass index (BMI) and body fat percentage (BFP). Anthropometric and biochemical measurements were carried out, and malondialdehyde serum levels were determined. Genotyping was done with the Axiom Genome-Wide LAT microarray, including 68 single nucleotide polymorphisms (SNPs) of the glutathione peroxidase (GPX) and paraoxonase (PON) families. We found six haplotypes associated with obesity—two of them (one in GPX3 and the other in GPX5 and GPX6) in a protective direction when obesity was classified by BMI. The other four haplotypes were associated with obesity when classification was based on BFP—one of them in GPX3 in a protective direction and the others in PON genes conferring obesity risk. In addition, two SNPs, GPX3 rs922429 and GPX4 rs2074451 showed protection against obesity classified by BFP. This study showed genetic susceptibility to oxidative stress in relation to obesity in Mexican children and opens up the possibility that some genetic loci related to obesity are not identified when weight classification is based on BMI.

## 1. Introduction

Childhood obesity has emerged as one of the most important public health problems in several countries. It is considered a strong predictor of adulthood obesity, particularly in cases of severe obesity and/or with a family history of obesity [1]. In 2016, the World Health Organization (WHO) estimated that nearly one in five children had overweight or obesity [2]. According to the Mexican National Health and Nutrition Survey, in 2018, the prevalence of overweight in children (5–11 years old) and adolescents (12–19 years old) was 18.1% and 23.8%, while the prevalence of obesity was 17.5% and 14.6%, respectively, keeping Mexico as the country with the highest prevalence of childhood obesity in the world [3]. Our group has reported that childhood obesity prevalence by body mass index (BMI) is underestimated by around 50% compared with the classification of obesity by body fat percentage (BFP) [4]. Therefore, it would be valid to suggest that many of the risk factors related to childhood obesity could have been underestimated when BMI was the primary method for obesity diagnosis.

Low-grade inflammation, hypoxia, and oxidative stress are well recognized underlying mechanisms of the pathogenesis of obesity and result from the imbalance between oxidant and antioxidant systems with a predominance of the oxidative activity [5,6]. Obesity can induce systemic oxidative stress through multiple biochemical mechanisms that include the mitochondrial respiratory chain; peroxisomal fatty acid metabolism; cytochrome P450 microsomal reactions; and several metabolic factors, such as dyslipidemia, hyperleptinemia, low antioxidant defense in adipose tissue, and chronic inflammation. Interestingly, oxidative stress could trigger obesity by stimulating the deposition of white adipose tissue and altering food intake; in turn, obesity can lead to oxidative stress, giving rise to a feedback loop [5,7].

Oxidative stress increases the lipid peroxidation rate during obesity due to elevated serum lipid concentrations and inversely correlates with high-density lipoprotein cholesterol (HDL) levels. Malondialdehyde (MDA) is one of the end products of the lipoperoxidation process and is the most frequently used oxidative stress biomarker [8,9]; however, results regarding oxidative stress biomarkers in childhood obesity are controversial. In some studies, MDA levels were significantly increased in children with obesity compared with non-obese children [9,10], while other reports suggest that increased activity of antioxidant systems in early obesity can counteract oxidative damage [11,12]. A stationary obesity state could lower tissue antioxidant defenses, as noticed by the depletion of glutathione (GSH) in the adipose tissue of children with obesity [13]. This decreased GSH availability could limit the ability of GSH peroxidase (GPx) to reduce lipid hydroperoxides into their corresponding alcohols and free hydrogen peroxides into water [14]. In addition, genetic variants of GPx and its isoforms have been associated with oxidative stress, obesity, and metabolic syndrome in human studies [15,16].

Another group of relevant antioxidant enzymes associated with obesity is the paraoxonase family (PON1, PON2, PON3), among which PON1 is the most studied one. PON1 is an HDL-associated enzyme that detoxifies the oxon derivatives of some organophosphate pesticides and is also involved in the prevention of lipid peroxidation [17]. Not only is its activity significantly reduced in children with metabolic syndrome and obesity [18,19], but some of its genetic variants were associated with childhood obesity in Spanish prepubertal children [15,20].

In this work, we analyzed genes from the GPx and PON families because they belong to the main antioxidant enzyme groups involved in the reduction of hydrogen peroxide and lipid hydroperoxides, and in the prevention of lipid peroxidation, respectively. Since both families have been poorly studied in the Mexican population, the current study aimed to contribute to the knowledge of the genetic influence of oxidative stress on the susceptibility to obesity in Mexican children and adolescents through the analysis of the associations of several GPx and PON SNPs with overweight and obesity.

## 2. Materials and Methods

### 2.1. Study Design

We conducted a case-control study including children and adolescents aged 3 to 17 years, from the Childhood Obesity Cohort-Healthy Childhood Project (COIPIS [Cohorte de Obesidad Infantil-Proyecto Infancia Saludable]) from the Hospital Regional Lic. Adolfo López Mateos, ISSSTE (Instituto de Seguridad y Servicios Sociales de los Trabajadores del Estado) [4]. This project was approved by the Ethics Committee of the Regional Hospital Lic. Adolfo López Mateos, ISSSTE, México (number 247.2012). All parents of the participants provided written informed consent prior to their inclusion in the study.

### 2.2. Clinical and Anthropometric Measurements 

Anthropometric measurements of weight, height, body mass index (BMI), and body fat percentage (BFP) were evaluated. The BFP was evaluated by electrical bioimpedance using a body composition analyzer with an accurate stadiometer (InBody J10, Korea). A whole blood sample was obtained to evaluate biochemical parameters after a 10-h fast: glucose, glycated hemoglobin (HbA1c), creatinine, triglycerides, total cholesterol, HDL cholesterol, and low-density lipoprotein (LDL) cholesterol. Concentrations were measured in an automated analyzer (Miura 200, Italy) and expressed as mg/dL, except forHbA1c, which was expressed as a percentage.

### 2.3. Malondialdehyde Quantification

A subsample of 165 children was considered for the quantification of serum MDA due to the availability of the sample. The product of the reaction of MDA in the sample with 1-methyl-2-phenylindole was measured at a wavelength of 586 nm [21]. Briefly, 200 μL of serum were mixed with 650 μL of 10 mM 1-methyl-2-phenylindole in acetonitrile/methanol (3:1 ratio); the reaction was started by adding 150 µL of hydrochloric acid (37%) and immediately incubated at 45 °C for 40 min. Finally, samples were centrifuged at 15,000× *g* for 5 min at room temperature, and the supernatant absorbance was determined at 586 nm and interpolated in a calibration curve consisting of six different concentrations of tetramethoxypropane (from 0.74 to 24 µM). Serum MDA concentration was expressed in µM.

### 2.4. DNA Isolation and Genotyping

Genomic DNA was obtained from 200 μL of whole blood using the commercial kit QIAsymphony DNA Minikit (QIAGEN, Hilden, Germany) by an automated nucleic acid isolation method (QIAsymphony SP/AS; Hilden, Germany). Genotyping was carried out using an automated genotyping equipment (GeneTitan, Affymetrix, ThermoFisher Scientific) with the Axiom Genome microarray Wide LAT (ThermoFisher Scientific). Genotype data were collected from the Axiom Suite program database (Applied, Biosystems). A total of 68 SNPs from several GPX and PON isoforms were chosen for this study (Table S1), based on the Axiom^®^ Genome-Wide LAT 1 Array. Additionally, minor allele frequency allele (MAF) was greater than 0.05 for this population of Mexican descendants, according to the 1000 Genomes Project.

### 2.5. Statistical Analysis

Participants were allocated as cases or controls based on two different criteria to determine obesity: (a) for BMI classification in obesity was done following the WHO cut-off in relation to sex and age (OB) [22,23,24]; (b) BFP cut-offs of 30% and 25% for girls and boys, respectively, for OB associated with low high-density lipoprotein (HDL), high triglycerides, high total cholesterol, high blood pressure, and cardiovascular disease [25,26], as previously reported in other COIPIS study [4].

When descriptive variables followed a Gaussian distribution, they were presented as means ± standard deviations (SD) and pairwise comparisons between case and control groups were made by a Student’s t-test. When the data did not follow a Gaussian distribution, they were presented as median and interquartile range, and the groups were compared using the Mann–Whitney U test in STATA12 (StataCorp, College Station, TX, USA).

We performed the exact test for the Hardy–Weinberg equilibrium (H–W), using the PLINK version 1.9 software (http://pngu.mgh.harvard.edu/purcell/plink Cambridge, MA, USA)[27]. We used Haploview 4.2 software (https://www.broadinstitute.org/haploview/haploview, Broad Institute Cambridge, MA, USA) to estimate the linkage disequilibrium (LD) measurement and to perform haplotype analysis, which included the plotting of haplotype block structure and testing of haplotype obesity associations [28]. We used the two-sided χ2 test to evaluate the differences in frequencies between case and control groups (unadjusted *p* < 0.05). In addition to haplotype association analyses, we used logistic regression to test associations among SNPs and obesity status for each of the two criteria to classify obesity and adjust by gender and age.

We tested each gene as an independent hypothesis; thus, Bonferroni correction for multiple comparisons was applied to each group of SNPs from the same gene, yielding the following *p* values: *p* < 0.003 for GPX3, *p* < 0.008 for GPX4, *p* < 0.008 for GPX5, *p* < 0.001 for GPX6, for GPX7, *p* < 0.002 for PON1, *p* < 0.002 for PON2, and *p* < 0.025 for PON3. Statistical power was estimated for a case-control, gene-only design, and the allele frequencies reported in Table S2, using the Quanto software version 1.2.4 (University of Southern California, Los Angeles, CA; http://biostats.usc.edu/Quanto.html). We considered the obesity prevalence reported in the ENSANUT 2018 [3]. Our study had 80% statistical power to detect OR ≤ 0.6, OR ≥ 1.5 with an allele risk frequency of 0.10. As allele frequency increased to 0.20 and 0.40, OR ≤ 0.7, OR ≥ 1.4, and OR ≤ 0.5, OR ≥ 1.35 could be detected.

## 3. Results

In this study, we included 585 children and adolescents aged 3 to 17 years (girls = 307; boys = 278). Descriptive data from cases and controls based on BMI and BFP criteria are shown in Table 1. The highest number of children classified as cases was found when the BFP cut offs of 30% and 25% for girls and boys (*n* = 297, 50.7%) were used, followed by BMI (*n* = 171, 29.2%). 

Significantly higher lipid profile values were found regardless of the obesity classification criterion, but no differences were found for glucose, creatinine, and MDA. Higher Hba1c levels were found for cases classified by BFP but not by BMI.

No deviation from H–W was found among SNPs. We found 13 blocks in LD; two of them (block 11 in GPX3, chromosome 5; and block 4 in PON1, chromosome 7) were in high LD (D′ > 0.8), and four blocks (5, 6, and 7 in PON1, chromosome 7; and block 8 in PON2 and PON3, chromosome 7) were in moderate LD (0.79 > D′ > 0.40). The seven blocks remaining were in low LD (D′ ≤ 0.39) (Table S1, Figure S1). 

Six haplotypes were significantly associated with obesity; two of them (one in GPX3 and the other one in GPX5 and GPX6) showed this association in a protective direction when obesity was classified by BMI. The other four haplotypes were associated with obesity when it was classified by BFP; one of them in GPX3, two others in PON1, and the last one in PON2 and PON3 (Table 2). Haplotypes in PON genes were associated in an increasing risk direction, while the one in GPX3 displayed a protective association.

The logistic regression model showed that when obesity was classified based on BFP, it was associated with eight SNPs in GPX3, GPX4, and PON1; however, the association remained in only one of them and was marginally significant for another one after Bonferroni adjustment: GPX4 rs2074451 and GPX3 rs922429, respectively. Importantly, both loci showed protection against obesity: GPX3 rs922429 T allele and GPX4 rs2074451 T allele (Table 3). No association between SNPs and obesity was found when the classification was based on BMI (*p* > 0.05).

## 4. Discussion

In this study, we analyzed the association of 68 SNPs in GPx and PON genes with obesity in Mexican children and adolescents. We found two haplotypes associated with obesity by BMI (in GPX3 and in GPX5 and GPX6) and five haplotypes associated with obesity by PBF (in GPX3, in PON1, and in PON2 and PON3). In addition, we found two polymorphisms the GPX4 rs2074451 and GPX3 rs922429 significantly and marginally associated with obesity by PBF respectively. No SNPs were found associated with obesity by BMI.

Concerning biochemical parameters, we found an altered lipid profile when either BMI or BFP was used to classify obesity, while higher HbA1c levels were found in the obesity group only when BFP was the classification criterion. These results are in agreement with the previous studies in Mexican children reported by Aradillas-García et al. [29] and Peralta-Romero et al. [30], where BMI was also the obesity diagnosis criterion; they found that glucose, total cholesterol, LDL cholesterol, and triglycerides were significantly higher and that HDL was lower in the obesity group compared with the normal-weight group.

Increased circulating lipids has been associated with an elevated rate of lipid peroxidation in the obesogenic state [31]; thus, it was important to evaluate oxidative damage by determining final products of lipid peroxidation such as MDA. Previous reports showed that MDA levels were significantly higher in children with obesity compared to those without obesity [9,10]; however, we did not find significant differences in the levels of MDA between the study groups. Other studies suggest that antioxidant enzyme activity is increased in early obesity to prevent oxidative stress [11,12]. We suggest that a mechanism to explain the lack of difference in lipoperoxidation between groups could be the increased activity of the antioxidant defense, as shown in the study by Codoñer-Franch et al. [8], where GPx activity was higher in children with obesity and inversely associated with total cholesterol. We did not evaluate GPx activity because plasma samples were not available for all children.

Interestingly, we found that two haplotypes in GPX3, GPX5, and GPX6, and two loci in GPx3 and GPx4 (the SNP in GPX3 did not belong to GPX3 haplotype) were protective factors against childhood obesity. Antioxidant activity of the GPx is ubiquitous: GPx3 is found in plasma, GPx4 is expressed in almost all cells and possesses a high affinity for lipid hydroperoxides, and GPx5 is found in the epididymis [32]. The rs922429 GPx3 variant had not been previously studied, so this is the first study showing association with obesity as a protective factor. Additionally, the rs2074451 GPX4 variant was associated with obesity as a protective factor and was in LD with rs757228 in block 1 (Table S1). The rs2074451 is a 3′UTR variant of the GPX4 gene which increase GPx activity. As was observed in a study with Spanish children (aged 3 to 13 years), the rs757228 GPX4 variant was found to be protective factor against obesity and the rs2074451 was associated with increased GPx activity in erythrocytes [15], so our results are in agreement with this previously reported evidence, suggesting a similar effect in this admixed Mexican children population. 

The PON family has several SNPs that may increase the risk of developing obesity [33]. In this research, three haplotypes; two in PON1, and one PON2 and PON3, were found to be risks factor for obesity. Two SNPs, rs854571 and rs13236941, belonging to the haplotypes in block 6 and 7, respectively, are 5′ upstream variants in the promoter region of PON1, but only in functional studies was the rs854571 (−832G > A) previously associated with increased PON1 protein levels and atorvastatin–lactone hydrolysis [34]. Regarding rs854571, a potential protective role for the C allele has been reported against hemorrhagic stroke in an adult Chinese population [35] and against cardiovascular risk in healthy adults from Italy consuming a high polyphenol and anthocyanin diet [36]. Our results showed that rs854570–rs854571 AC haplotype is a risk factor for childhood obesity. The reported results and ours are not necessarily contradictory: they come from different (but associated) diseases, outcomes, ancestry, and age. In fact, in an admixture, like the Mexican population, the alleles frequency may change with respect to the parental population changing the pattern of LD, giving a different association (i.e., size effect and direction) [37]. Besides, our results came from a haplotype instead of a single polymorphism. Regarding the rs13236941 variant, although there are no previous reports related about a risk-or protection effect relationship, our results suggest that, together with the rs757158 variant (rs13236941–rs757158 CC haplotype) it is a risk factor for obesity and a relevant target for future research regarding nutraceutical treatments.

The most studied PON1 polymorphism is the rs662 (also named Q192R), which has been associated with higher abdominal circumference, BFP, and BMI Z-scores in school-age children from Denmark prenatally exposed to pesticides [38]. In a study with 117 Mexican children 6–12 years old, the PON1 rs662 polymorphism was associated with insulin resistance but not with overweight or obesity [39], in agreement with our study. 

This study has sufficient statistical power (80%) to demonstrate the associations of the GPX3 rs922429 and GPX4 rs2074451 variants with childhood obesity; however, it has some limitations such as the sample size and the plasma availability to evaluate GPx activity. First of all, the lack of significance of several of the gene variants could not be because of a lack of statistical power to detect a low effect in a low sample size. We suggest validating the associations of the SNP whose significance was lost with the Bonferroni correction in a larger and independent sample. In addition, given that our results of these two families of antioxidant enzyme genes (GPx and PON1) show an association between the antioxidant defense and protection against and risk for obesity, it would be advisable to analyze a larger set of antioxidant enzyme genes to get a more complete picture of this relationship.

An important point to highlight from this study is that it was possible to find SNPs in non-coding regions by considering more than just the most studied polymorphisms located in coding regions. Besides, the fact that the classification of obesity by BFP instead of BMI allowed finding several associations with the polymorphisms studied here opens the possibility of the existence of other genetic loci associated with obesity that could not have been identified by the BMI criterion in other studies.

## 5. Conclusions

In this study we found six haplotypes associated with obesity, two of them (one in GPX3 and the other in GPX5 and GPX6) when obesity was classified by BMI. The other four haplotypes were associated with obesity when classification was based on BFP—one of them in GPX3 in a protective direction and the others in PON genes. In addition, we identified a protective effect of SNPs in GPX3 rs922429 and GPX4 rs2074451 in Mexican children and adolescents, only when obesity was defined under the BFP criterion instead of BMI.

## Figures and Tables

**Table 1:** Descriptive characteristics of Mexican children cases and controls classified according to their body mass indexes (BMIs) or body fat percentages (BFPs).

	BMI			BFP		
Variables	Cases	Control	*p*	Cases	Control	*p*
*n* (%)	171 (29.2)	414 (70.8)		306 (52.3)	279 (47.7)	
Boys *n* (%)	108 (63.1)	200 (48.3)	0.0014	186 (60.2)	124 (44.4)	<0.001
Age (years)	10.8 (7.0–13.6)	10.3 (7–13.3)	0.57	11.1 (7.8–13.3)	9.9 (6.8–13.4)	0.070
BFP	40.7 (35.8–45.0)	23.7 (18.3–28.7)	0.0001	35.2 (30.2–41.8)	20 (16.1–24.5)	<0.001
BMI (kg/m^2^)	24.1 (19.1–27.0)	17.9 (16.2–20.4)	0.0001	21.69 (18.2–25.3)	16.9 (15.5–19.5)	<0.001
Glucose (mg/dl)	97.5 (91.0–104.0)	97 (90–103)	0.55	98.13 ± 9.1	96.33 ± 10.7	0.050
HbA1c (%)	5.5 (5.3–5.7)	5.5 (5.2–5.6)	0.17	5.5 (5.3–5.7)	5.4 (5.1–5.6)	0.009
Creatinine (mg/dl)	0.98 (0.81–1.16)	0.94 (0.82–1.11)	0.39	0.98 (0.8–1.2)	0.92 (0.81–1.10)	0.080
Triglycerides (mg/dl)	110 (84–160)	93 (70–120)	0.0001	105 (83–147)	86 (67–117)	<0.001
Total colesterol (mg/dl)	171 (152–191)	162 (142–181)	0.01	167 (149–188)	161 (141–180)	0.010
HDL-cholesterol (mg/dl)	57.64 ± 11.8	61.82 ± 12.20	0.0008	58.85 ± 12.13	62.45 ± 11.86	0.002
LDL-cholesterol (mg/dl)	92.8 (78.9–108.9)	87.1 (71.4–100.5)	0.002	90.7 (77.7–105.2)	86.6 (70.5–100.6)	0.007
Malondialdehyde (µM)*	2.20 (1.8–3.6)	2.98 (1.9–3.9)	0.17	2.6 (1.87–3.82)	2.9 (1.8–4.0)	0.960

Values are expressed as means ± SD or median (interquartile range). BMI (body mass index), BFP (body fat percentage), HbA1c (glycated hemoglobin), *p* (*p*-value), *n* (number of children). * Malondialdehyde analysis was performed on a subsample of 165 children.

**Table 2:** Haplotypes associated with body mass index (BMI) and body fat percentage (BFP).

	**Haplotypes Associated with Obesity by BMI**							
**Gene**	**Block Number**	**SNP**	**Block**	**F**	**CC F**	**Χ^2^**	**OR**	***p***
GPX3	13	rs3792798						
		rs3828599	GGGATCC	0.59	0.59, 0.58	0.2	1.06	0.68
		rs8177427	AAGGGTT	0.26	0.24, 0.25	0.4	0.91	0.54
		rs8177431	GAAGGCT	0.08	0.09, 0.07	1.0	1.27	**0.32**
		rs8177435	GGGGGCT	0.05	0.02, 0.05	5.8	0.36	**0.02**
		rs11548						
		rs736775						
GXP5 and	12	rs35921765	ACATAAAA	0.54	0.47, 0.56	6.8	0.70	**0.01**
GPX6		rs9468385	CTCCGCGC	0.30	0.33, 0.28	2.3	1.25	**0.13**
		rs406113	ACCCAAGA	0.06	0.06, 0.06	0.2	1.13	0.66
		rs1003359	ACCCACGC	0.03	0.04, 0.03	0.9	1.40	0.34
		rs2064424	ACCCAAAC	0.02	0.03, 0.02	1.3	1.61	0.25
		rs440481	ATCCACGA	0.01	0.01, 0.02	0.9	0.49	0.34
		rs451774	ACCTAAGA	0.01	0.01, 0.01	0.6	1.62	0.43
		rs62402376						
	**Haplotypes Associated with Obesity by BFP**							
**Gene**	**Block Number**	**SNP**	**Block**	**F**	**CC F**	**Χ^2^**	**OR**	***p***
GPX3	12	rs841236	AGGT	0.66	0.66, 0.65	0.1	1.04	0.765
		rs707144	GAAG	0.24	0.24, 0.23	0.0	0.99	0.959
		rs707145	GAGT	0.04	0.04, 0.03	0.5	1.25	0.475
		rs707148	AAGT	0.04	0.02, 0.04	4.7	0.49	0.030
PON1	6	rs854570	AC	0.57	0.61, 0.52	8.2	1.40	0.004
		rs854571	CT	0.40	0.36, 0.44	7.8	0.72	0.005
			CC	0.03	0.02, 0.03	0.1	0.90	0.750
PON1	7	rs757158	CC	0.47	0.50, 0.43	4.5	1.28	0.034
		rs13236941	TC	0.21	0.21, 0.21	0.1	1.04	0.789
PON2 and PON3	8	rs3757708	GCAG	0.48	0.47, 0.48	0.3	0.94	0.614
		rs2072200	TGGG	0.28	0.28, 0.28	0.0	1.00	0.994
		rs17881071	GGGC	0.20	0.20, 0.19	0.2	1.07	0.631
		rs7493	GGGG	0.01	0.02, 0.01	4.3	3.54	0.038

Number haplotype block (block), frequency of haplotype (F), case and control frequency of the haplotype (CC F), chi square value (X^2^), odds ratio (OR), *p*-value (*p*).

**Table 3:** Associations between obesity (classified according to percentage of body fat) and antioxidant enzyme-related single nucleotide polymorphisms (SNPs) in Mexican children.

CHR	Gene	SNP	A	Model	OR (IC95%), *p*	P_Bonf_
5	GPX3	rs922429	T	ADD	0.69 (0.53–0.90), 0.005	0.003
		rs922429	T	REC	0.40 (0.21–0.73), 0.003	0.003
19	GPX4	rs713041	T	REC	0.56 (0.32–0.95), 0.033	0.008
19	GPX4	rs2074451	T	REC	0.57 (0.33–0.97), 0.004	0.008
7	PON1	rs13236941	T	ADD	0.73 (0.57–0.95), 0.020	0.002
		rs13236941	T	DOM	0.68 (0.49–0.95), 0.024	0.002
7	PON1	rs3917558	C	ADD	0.76 (0.58–1.00), 0.049	0.002
7	PON1	rs854555	C	REC	1.71 (1.12–2.62), 0.013	0.002
7	PON1	rs854571	T	ADD	0.73 (0.57–0.94), 0.015	0.002
		rs854571	T	DOM	0.66 (0.46–0.93), 0.018	0.002
7	PON1	rs854569	T	ADD	0.77 (0.60–0.99), 0.042	0.002

Chromosome (CHR), associated allele (A), inheritance model (ADD: additive, DOM: dominant, and REC: recesive), odds ratio (OR), interval confidence at 95% (IC95%), *p*-value (*p*), *p*-value after Bonferroni adjustment by number of SNPs by gene (P_Bonf_).

## Supplementary Materials

The following are available online at https://www.mdpi.com/2076-3921/9/8/684/s1. Figure S1: Linkage disequilibrium and association plot of GPX3, GPX4, GPX5, GPX6, GPX7, PON1, PON2, and PON3 in Mexican children. (A) Block 1 in GPX4, block 2 in GPX5 and GPX6, and block 3 in GPX7. (B) Blocks 4 to 7 in PON1, block 8 in PON3, and blocks 9 and 10 in PON2. (C) Blocks 11 to 13 in in GPX3, Table S1: Selected SNPs in GPX and PON genes; chromosome (Chr), minor allele (Allele), minor allele frequency (MAF), linkage disequilibrium block (LD blocks), and multiallelic D prime (D′).
