# Supplementary material for: Antioxidant Enzymes Haplotypes and Polymorphisms Associated with Obesity in Mexican Children
Source: Antioxidants (Basel). 2020 Aug 1;9(8):684. doi: 10.3390/antiox9080684 (PMC7464274; doi:10.3390/antiox9080684)
Supplement: Supplementary file 1 [file antioxidants-09-00684-s001.pdf]

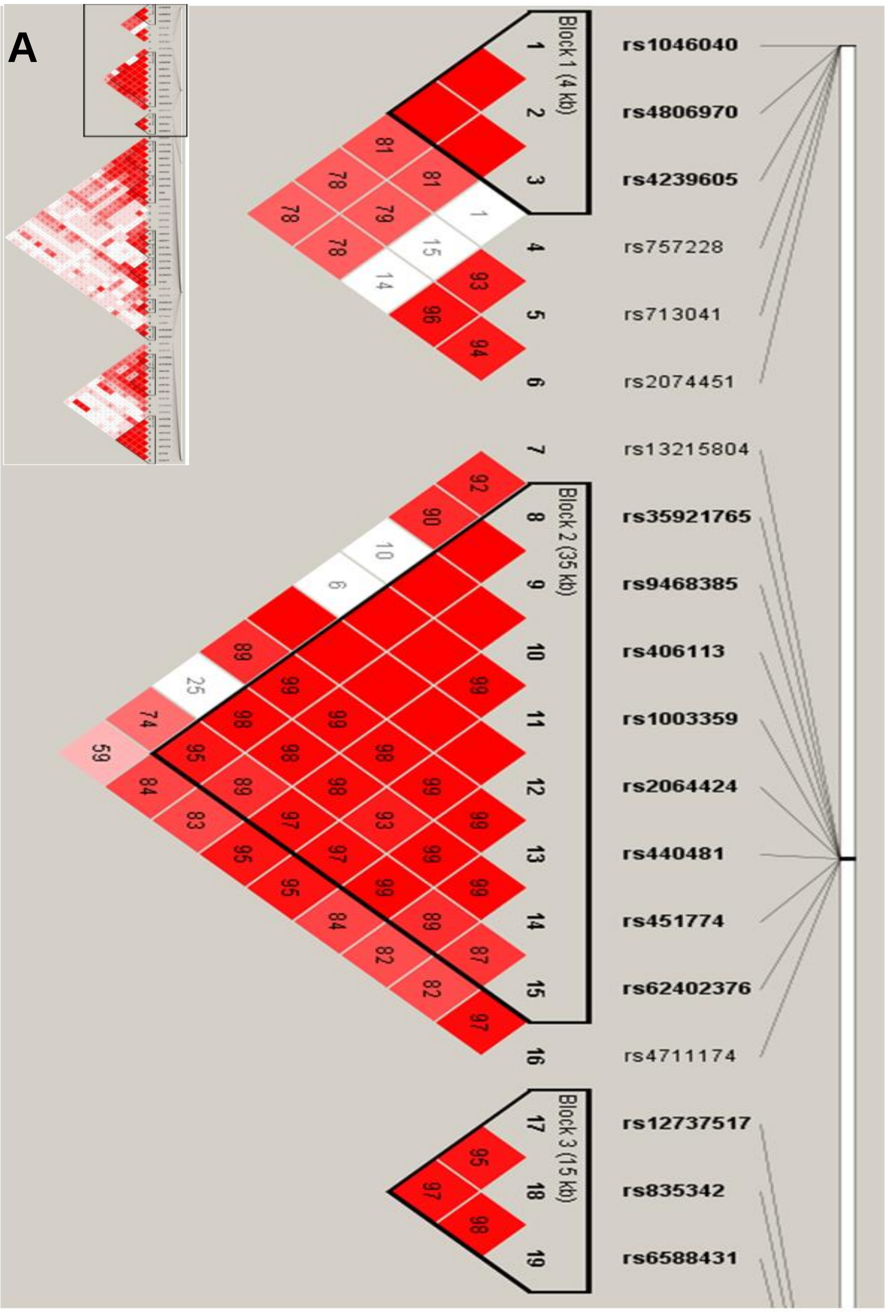

A
Block 1 (4 kb)
Block 2 (35 kb)
Block 3 (15 kb)
rs1046040
rs4806970
rs4239605
rs757228
rs713041
rs2074451
rs13215804
rs35921765
rs9468385
rs406113
rs1003359
rs2064424
rs440481
rs451774
rs62402376
rs4711174
rs12737517
rs835342
rs6588431

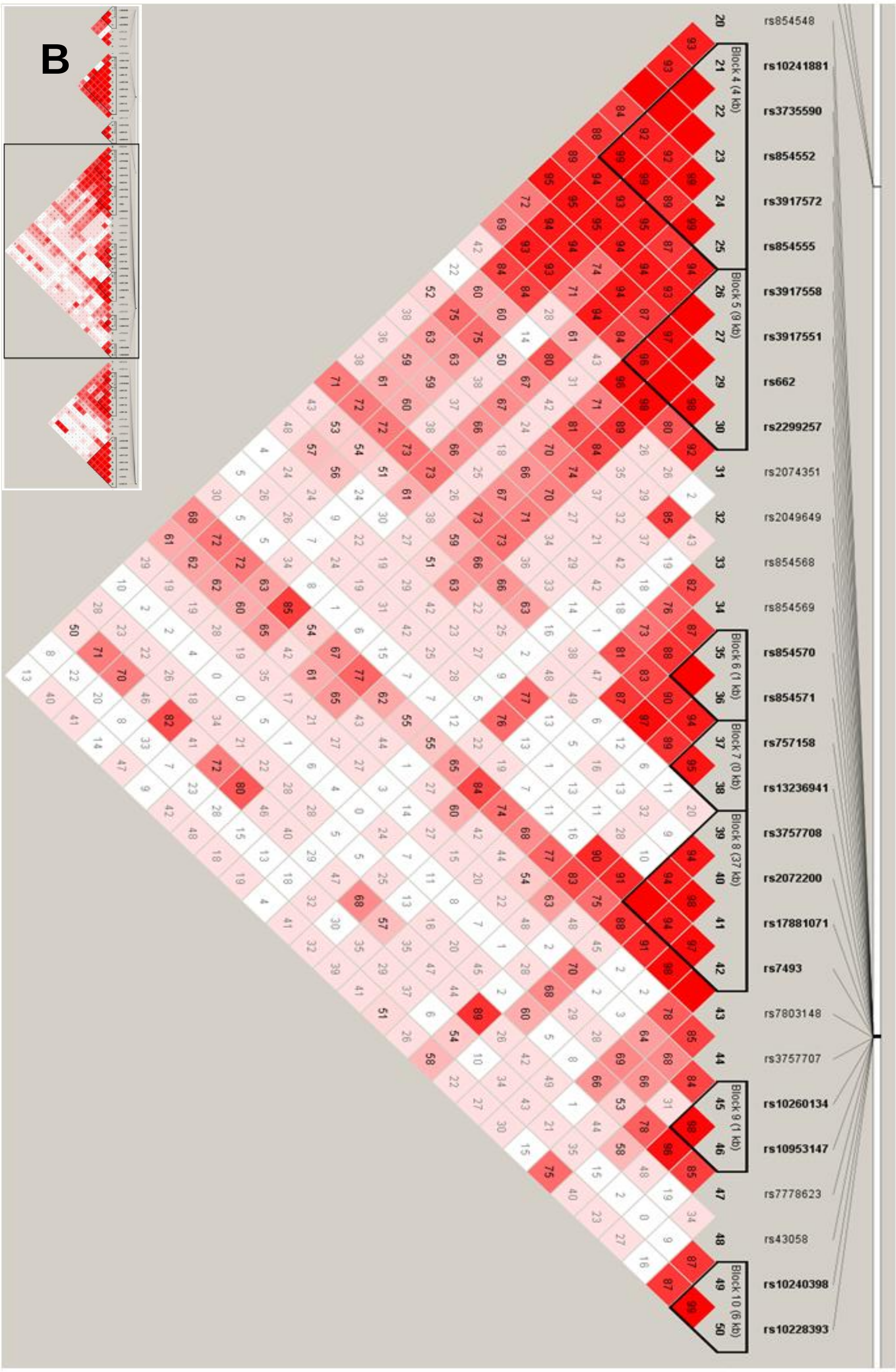

B
Block 4 (4 kb)
Block 5 (9 kb)
Block 6 (1 kb)
Block 7 (0 kb)
Block 8 (37 kb)
Block 9 (1 kb)
Block 10 (6 kb)
rs854548
rs10241881
rs3735590
rs854552
rs3917572
rs854555
rs3917558
rs3917551
rs662
rs2299257
rs2074351
rs2049649
rs854568
rs854569
rs854570
rs854571
rs757158
rs13236941
rs3757708
rs2072200
rs17881071
rs7493
rs7803148
rs3757707
rs10260134
rs10953147
rs7778623
rs43058
rs10240398
rs10228393

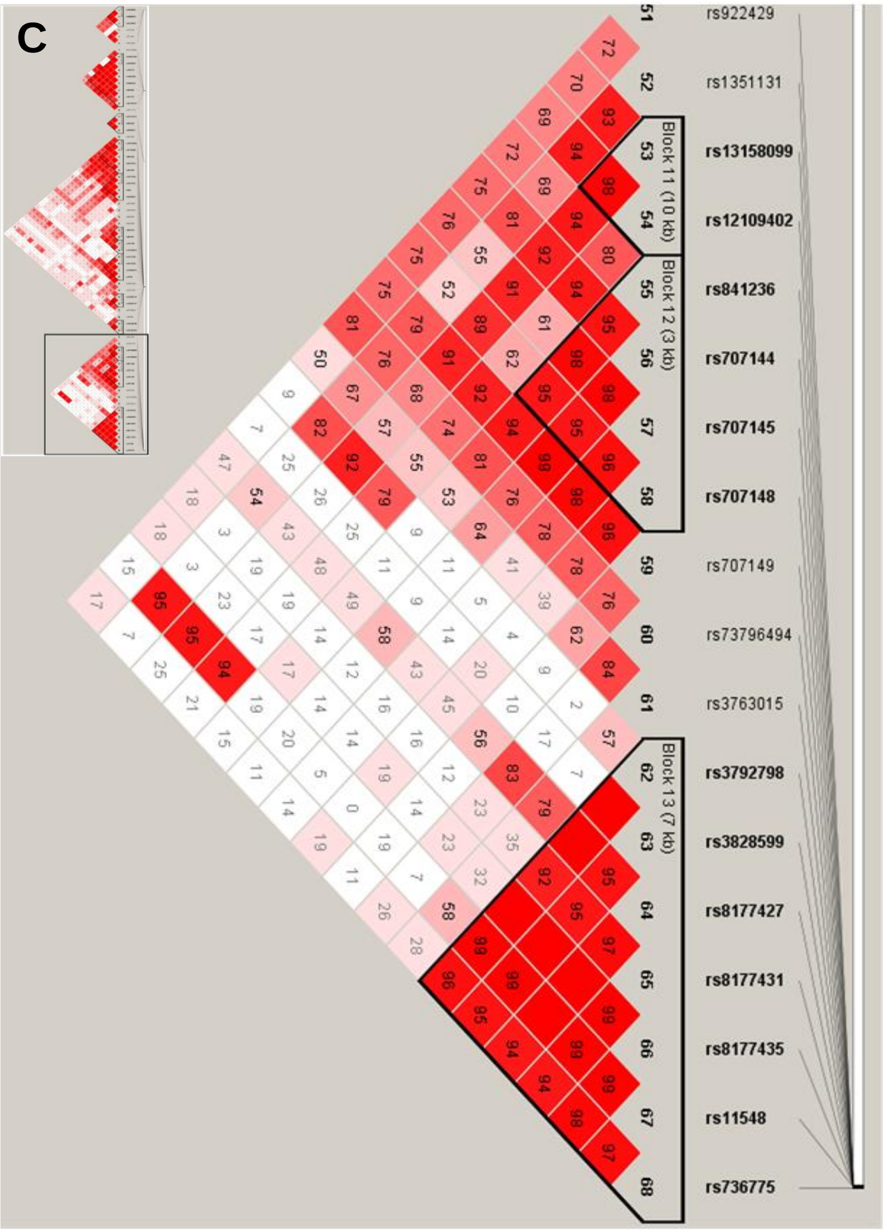


Figure S1. Linkage desequilibrium and association plot of GPX3, GPX4, GPX5,GPX6, GPX7, PON1, PON2, and PON3 in Mexican children. A) Block 1 in GPX4, Block 2 in GPX5 and GPX6, and Block 3 in GPX7. B) Blocks 4 to7 in PON1, Block 8 inPON3, and Blocks 9 and 10 in PON2. C) Blocks 11 to 13 in GPX3.

Table S1. Selected SNPs in GPX and PON genes. Chromosome (Chr), minor allele (Allele), minor allele frequency (MAF), linkage disequilibrium blocks (LD Blocks), and multiallelic D prime (D´).

| Gene | Chr | SNP | Allele | MAF | LD Blocks | D´ |
|---|---|---|---|---|---|---|
| GPX3 | 5 | rs13158099 | G | 0.1824 | Block 11 | 0.91 |
| | 5 | rs12109402 | C | 0.2441 | Block 11 | |
| | 5 | rs3763015 | C | 0.2382 | | |
| | 5 | rs707144 | A | 0.3224 | Block 12 | 0.2 |
| | 5 | rs841236 | G | 0.2943 | Block 12 | |
| | 5 | rs707145 | A | 0.2449 | Block 12 | |
| | 5 | rs707148 | G | 0.2552 | Block 12 | |
| | 5 | rs73796494 | T | 0.4203 | | |
| | 5 | rs707149 | G | 0.3185 | | |
| | 5 | rs1351131 | G | 0.2732 | | |
| | 5 | rs922429 | T | 0.315 | | |
| | 5 | rs8177427 | A | 0.0832 | Block 13 | Reference |
| | 5 | rs736775 | T | 0.3941 | Block 13 | |
| | 5 | rs11548 | T | 0.2568 | Block 13 | |
| | 5 | rs8177431 | G | 0.4005 | Block 13 | |
| | 5 | rs8177435 | G | 0.4122 | Block 13 | |
| | 5 | rs3828599 | A | 0.3592 | Block 13 | |
| | 5 | rs3792798 | A | 0.2794 | Block 13 | |
| GPX4 | 19 | rs4239605 | A | 0.276 | Block 1 | 0.067 |
| | 19 | rs4806970 | A | 0.4451 | Block 1 | |
| | 19 | rs1046040 | A | 0.4476 | Block 1 | |
| | 19 | rs757228 | A | 0.3631 | Block 1 | |
| | 19 | rs2074451 | T | 0.3346 | Block 1 | |
| | 19 | rs713041 | T | 0.3352 | Block 1 | |
| GPX5 | 6 | rs2064424 | G | 0.2978 | | |
| | 6 | rs451774 | G | 0.4309 | Block 2 | 0.11 |
| | 6 | rs2064424 | G | 0.33 | Block 2 | |
| | 6 | rs440481 | C | 0.3528 | Block 2 | |
| | 6 | rs9468385 | T | 0.3182 | Block 2 | |
| | 6 | rs1003359 | C | 0.4441 | Block 2 | |
| GPX6 | 6 | rs406113 | C | 0.4522 | Block 2 | |
| | 6 | rs35921765 | C | 0.3056 | Block 2 | |
| | 6 | rs13215804 | G | 0.2131 | | |
| | 6 | rs62402376 | C | 0.3586 | | |
| | 6 | rs4711174 | A | 0.269 | | |

| | | | | | | |
|---|---|---|---|---|---|---|
| **GPX7** | 1 | rs6588431 | T | 0.2763 | Block 3 | 0.09 |
| | 1 | rs835342 | G | 0.2984 | Block 3 | |
| | 1 | rs12737517 | T | 0.4403 | Block 3 | |
| **PON 1** | 7 | rs2299257 | A | 0.385 | Block 5 | 0.44 |
| | 7 | rs662 | T | 0.4074 | Block 5 | |
| | 7 | rs3917549 | A | 0.4271 | Block 5 | |
| | 7 | rs3917551 | A | 0.2555 | Block 5 | |
| | 7 | rs3917558 | C | 0.2707 | Block 5 | |
| | 7 | rs854555 | C | 0.4544 | Block 4 | 0.84 |
| | 7 | rs3917572 | G | 0.281 | Block 4 | |
| | 7 | rs3735590 | A | 0.2704 | Block 4 | |
| | 7 | rs10241881 | A | 0.2707 | Block 4 | |
| | 7 | rs854552 | C | 0.3995 | Block 4 | |
| | 7 | rs757158 | C | 0.4754 | Block 7 | 0.49 |
| | 7 | rs13236941 | T | 0.3218 | Block 7 | |
| | 7 | rs43058 | C | 0.1698 | | |
| | 7 | rs2049649 | A | 0.3274 | | |
| | 7 | rs854568 | G | 0.3425 | | |
| | 7 | rs2074351 | A | 0.3446 | | |
| | 7 | rs854569 | T | 0.4173 | | |
| | 7 | rs854548 | A | 0.3613 | | |
| | 7 | rs7778623 | G | 0.4015 | | |
| | 7 | rs854570 | C | 0.4347 | Block 6 | 0.74 |
| | 7 | rs854571 | T | 0.4039 | Block 6 | |
| **PON2** | 7 | rs10240398 | T | 0.2449 | Block 10 | 0.06 |
| | 7 | rs10228393 | C | 0.413 | Block 10 | |
| | 7 | rs10260134 | T | 0.3797 | Block 9 | 0.23 |
| | 7 | rs10953147 | A | 0.4327 | Block 9 | |
| | 7 | rs7493 | C | 0.2061 | Block 8 | 0.47 |
| | 7 | rs17881071 | A | 0.4851 | Block 8 | |
| **PON3** | 7 | rs2072200 | G | 0.4977 | Block 8 | |
| | 7 | rs3757708 | T | 0.2978 | Block 8 | |
